# Complete Penile Duplication with Structurally Normal Penises: A Case Report

**DOI:** 10.4274/balkanmedj.2017.1518

**Published:** 2018-07-24

**Authors:** Ahsen Karagözlü Akgül, Murat Uçar, Fatih Çelik, İrfan Kırıştıoğlu, Nizamettin Kılıç

**Affiliations:** 1 Clinic of Pediatric Urology, University of Health Sciences, Van Training and Research Hospital, Van, Turkey; 2 Clinic Pediatric Urology, University of Health Sciences, İzmir Tepecik Training and Research Hospital, İzmir, Turkey; 3 Department of Pediatric Surgery, Uludağ University School of Medicine, Bursa, Turkey; 4 Division of Pediatric Urology, Department of Pediatric Surgery, Uludağ University School of Medicine, Bursa, Turkey

**Keywords:** Congenital anomaly, diphallia, penile duplication, reconstructive surgery

## Abstract

**Background:**

Diphallia is a very rare anomaly and seen once in every 5.5 million live births. True diphallia with normal penile structures is extremely rare. Surgical management for patients with complete penile duplication without any penile or urethral pathology is challenging.

**Case Report:**

A 4-year-old boy presented with diphallia. Initial physical examination revealed first physical examination revealed complete penile duplication, urine flow from both penises, meconium flow from right urethra, and anal atresia. Further evaluations showed double colon and rectum, double bladder, and large recto-vesical fistula. Two cavernous bodies and one spongious body were detected in each penile body. Surgical treatment plan consisted of right total penectomy and end-to-side urethra-urethrostomy. No postoperative complications and no voiding dysfunction were detected during the 18 months follow-up.

**Conclusion:**

Penile duplication is a rare anomaly, which presents differently in each patient. Because of this, the treatment should be individualized and end-to-side urethra-urethrostomy may be an alternative to removing posterior urethra. This approach eliminates the risk of damaging prostate gland and sphincter.

Penile duplication or diphallia is a rare anomaly that may present as an isolated anomaly or concomitant with other systemic anomalies. The incidence of diphallia is 1 per 5-6 million live births. Since every case has unique features, diphallia represents a great challenge for reconstruction. Frequently, penile duplication presents with penile anomalies such as hypospadias, epispadias, and a rudimentary penis (1,2,3,4,5,6,7). Presentation of two completely developed structurally normal penises is extremely rare. Here, we report a case with two completely developed, structurally normal penises and its surgical management with penectomy and end-to-side urethra-urethrostomy.

## CASE PRESENTATION

**Figure 1:**
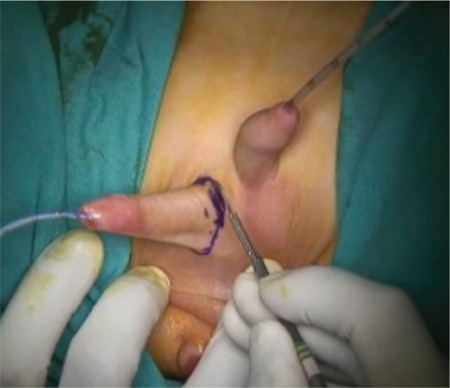
Preoperative appearance of true diphallia with urethral catheterization.

**Figure 2:**
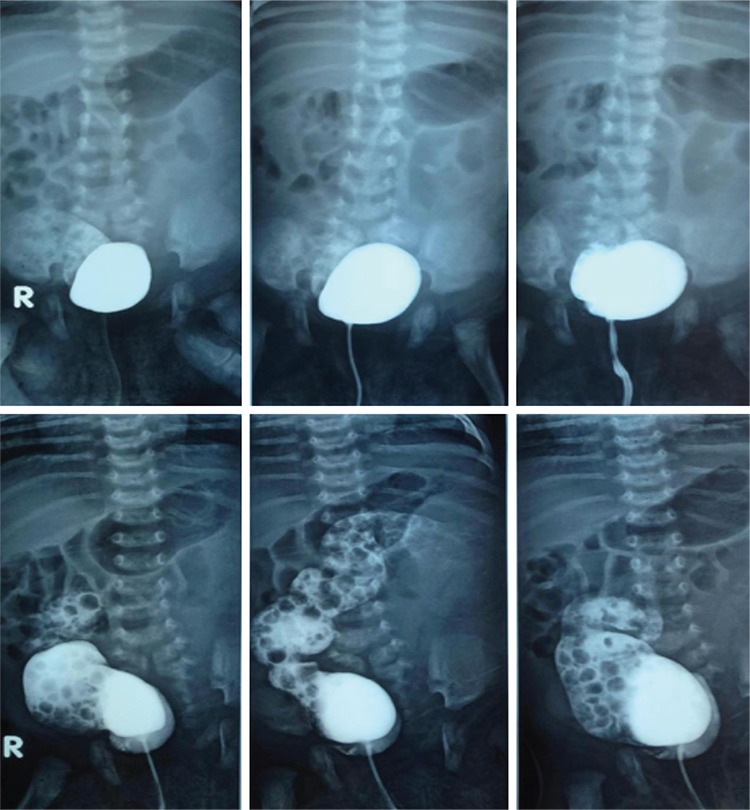
Cystography showing right bladder filled with contrast via right urethra and a large rectovesical fistula.

**Figure 3:**
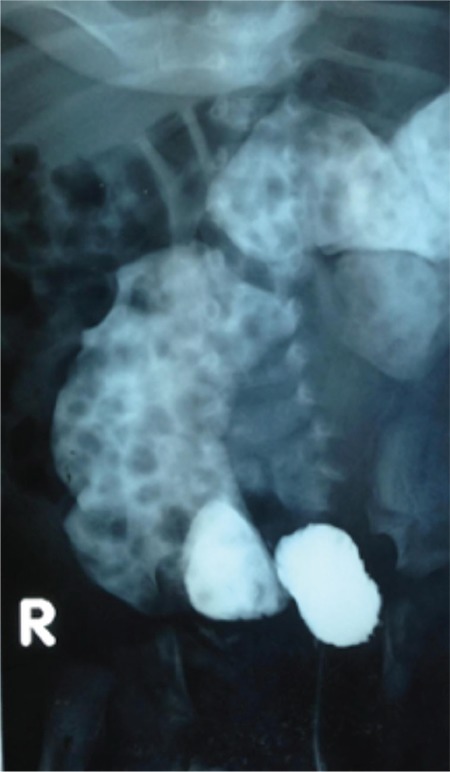
Cystography showing left bladder filled with contrast via left urethra.

A 4-year-old boy presented with diphallia (Figure 1). Initial physical examination revealed two completely developed penise with urine flow from both urethras and two anal dimples with anal atresia. Meconium flow from right urethra was observed upon closer examination. There was one testis on each side of the scrotum. On his first day of life, laparotomy was performed that showed duplicated rectum, colon, cecum, and appendix and duplication of 5 cm of terminal ileum. Ileostomy was performed from the proximal region of the duplicated bowel. Doppler ultrasonography (USG) showed two completely developed penises, two cavernous bodies, one spongious body, and normal vascular supply to each penis. Abdominal USG and magnetic resonance imaging (MRI) showed one kidney and one ureter on each side and two bladders. Right urethra was catheterized, and cystography revealed a large rectovesical fistula between the right colon and the right bladder (Figure 2). Second cystography was performed via left urethra and showed no abnormalities (Figure 3). MRI revealed two prostatic glands. Conventional urodynamic study was performed during the patient’s first year of life. Capacity of right half bladder was 50 mL and capacity of left side was 40 mL, while the normal expected bladder capacity is 63-70 mL. Compliances of half bladders were 8 and 7 cm H_2_O/mL, respectively.

Abdominoperineal pull-through was performed in patient’s first month of life. One of the right colons was removed after closing its fistula.

**Figure 4:**
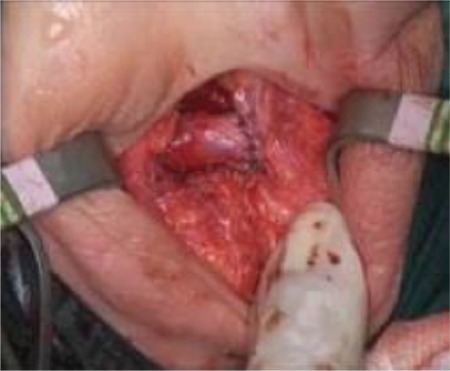
Right-to-left end-to-side urethral-urethrostomy (perineal vision).

**Figure 5:**
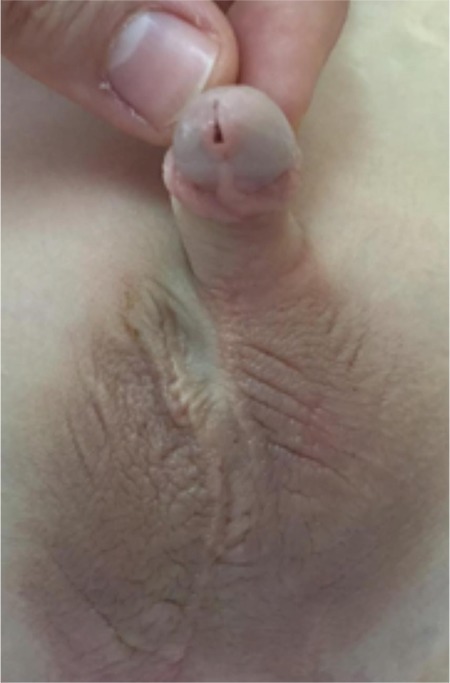
Visual appearance at postoperative 6 months.

When the patient was 4 years old, cystoscopy through each urethra, right total penectomy, and right-to-left end-to-side urethral-urethrostomy were performed (Figure 4). Cystourethroscopic examination revealed that both urethras, bladder necks, and bladders were normal. Because the left penis was closer to the midline, it was decided to perform right penectomy. After the dissection and excision of the two cavernous bodies, anterior urethra was dissected and removed. At this level, end-to-side urethra-urethrostomy was performed. Postoperative follow-up of 18 months was uneventful and without dilation of the upper urinary tract or any urinary tract infection (Figure 5). On observation, the patient’s voiding was normal. Informed consent was obtained from the parents of the patient.

## DISCUSSION

True diphallia is a rare anomaly with a wide spectrum of presentation due to associated anomalies. About 100 cases were reported in the literature and it is believed that no two are identical due to significant anatomical variety.

Recently accepted classification defines the following two major groups: true diphallia and bifid phallus. These two groups are further divided into partial or complete duplication. True complete diphallia is defined by complete penile duplication, each with two corpora cavernosa and one corpus spongiosum (8). If the duplicate penis is smaller or rudimentary with complete structures, it is described as a true partial diphallia. When there is only one corpus cavernosum in each penis, the term bifid phallus is used. Moreover, if the degree of separation is complete to the base of the shaft or just to the glans, the anomaly is described as complete or partial bifid phallus, respectively (8).

True diphallia is the less common variety and usually presents with a wider range of associated malformations such as bladder and urethral duplication, exstrophy vesica, renal anomalies, bifid scrotum, anorectal malformations, bowel duplication, and vertebral anomalies (8,9). Bifid phallus is usually associated with less severe malformations (8). True diphallia can rarely be isolated (2). Muramatsu et al. (7) reported about a case of a 15-year-old patient presenting with VATER syndrome, chronic renal failure, and penile duplication, including a hypoplastic lower urinary tract.

Penile duplication usually presents with associated penile anomalies such as hypospadias, epispadias, chordee, ectopic urethra, and blind-ended or hypoplastic urethra (1,2,3,4,5,6,7). In our case, true diphallia presented with multiple associated anomalies such as anal atresia and duplication of the bowel and bladder, but there was no pathology in the penile structures in both penises.

Penile duplication and multiple associated anomalies can be managed with multiple surgeries (treatment of the associated anomalies and genital reconstruction) (9). De Oliveira et al. (2) reported an isolated true partial diphallia that was managed in a single surgery with right penectomy and end-to-side anastomosis between the right urethra and the remaining urethra. Excision of the ventral penis and using its preputial skin for hypospadias repair (Duckett tube) of the dorsal penis has also been reported in the literature (3). Another case with imperforate anus, double bladder, and duplicated penis, reported by Mirshemirani et al. (4), was managed with laparotomy and colostomy on the third day of life, and cystoplasty and reimplantation of left ureter in a single bladder and resection of left phallus were performed when the patient was 4 months old. If there is one corpus cavernosum in each penis, joining two corporal bodies with penile reconstruction is preferred (1,6). Elsawy et al*.* (5) reported a joining technique without the removal of any penis even for true diphallia with two corpora in each penis. In the management of our case, the first surgery to correct concomitant anomalies was performed during the patient’s newborn period, and right penectomy and urethral-urethrostomy were performed at a later stage of his toddlerhood period. We did not want to leave a blind stump of right urethra that could increase the risk of infection. Because there was one testicle and one seminal vesicle on each side, we did not fully excise the right urethra, but we did not close it either. Therefore, we did not block the flow of seminal fluid from the right testis and did not block the way to reach the right prostate retrograde.

The wide variability of the anatomy and the presentation in diphallia cases result in different management strategies of the pathology. Surgical correction is individualized with the aim of achieving proper urinary continence and erection with adequate cosmesis. We believe that penectomy and urethral-urethrostomy without removing the posterior urethra may be a better choice for individualized treatment of such duplicated penises without the risk of damaging the sphincter and the prostate gland.

## Figures and Tables

**Figure of abstract:**
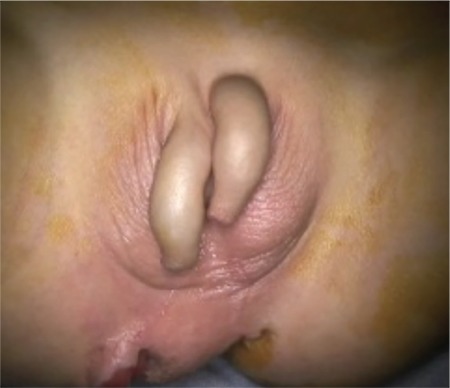
True diphallia with structurally developed penises.

## References

[1] Tirtayasa PM, Prasetyo RB, Rodjani A (2013). Diphallia with Associated Anomalies: A Case Report and Literature Review. Case Rep Urol.

[2] De Oliveira MC, Ramires R, Soares J, Carvalho AP, Marcelo F (2010). Surgical treatment of penile duplication. J Pediatr Urol.

[3] Bhat HS, Sukumar S, Nair TB, Saheed CS (2006). Successful surgical correction of true diphallia, scrotal duplication, and associated hypospadias. J Pediatr Surg.

[4] Mirshemirani A, Roshanzamir F, Shayeghi S, Mohajerzadeh L, Hasas-Yeganeh S (2010). Diphallus with Imperforate Anus and Complete Duplication of Recto Sigmoid Colon and Lower Urinary Tract. Iran J Pediatr.

[5] Elsawy M, Pippi Salle JL, Abdulsalam M, Alsaid AN (2012). Penile duplication: Is it necessary to excise one of the penises?. J Pediatr Urol.

[6] Corrêa Leite MT, Fachin CG, de Albuquerque Maranhão RF, Francisco Shida ME, Martins JL (2014). Penile duplication without removal of corporal tissue: Step by step of an excellent cosmetic result. J Pediatr Urol.

[7] Muramatsu M, Shishido S, Nihei H, Hamasaki Y, Hyodo Y, Kawamura T, et al (2015). Urinary reconstruction in vertebral, anorectal, cardiac, trachea-esophageal, renal abnormalities and limb defects association with chronic renal failure and penile duplication. Int J Urol.

[8] Gyftopoulos K, Wolffenbuttel KP, Nıjman RJ (2002). Clinical and embryologic aspects of penile duplication and associated anomalies. Urology.

[9] Karaca I, Turk E, Ucan AB, Yayla D, Itirli G, Ercal D (2014). Surgical management of complete penile duplication accompanied by multiple anomalies. Can Urol Assoc J.

